# Supplementary material for: MetaRibo-Seq measures translation in microbiomes
Source: Nat Commun. 2020 Jun 29;11:3268. doi: 10.1038/s41467-020-17081-z (PMC7324362; doi:10.1038/s41467-020-17081-z)
Supplement: Supplementary file 10 — Supplementary Data 7 [file 41467_2020_17081_MOESM10_ESM.zip › File2/Confidence_High_Taxonomy/366556_out.krona.html]

Javascript must be enabled to view this page.

members
magnitude
magnitudeUnassigned
count
unassigned
taxon
rank

366556\_out

1

1
superkingdom
2

32066
phylum
1

203490
1
class

203491
order
1

203492
family
1

848
genus
1

851
species
1


SRS148205\_contig\_number\_contig-100\_3390.147618
76857
1
subspecies
